# Celery (*Apium graveolens* L.) Performances as Subjected to Different Sources of Protein Hydrolysates

**DOI:** 10.3390/plants9121633

**Published:** 2020-11-24

**Authors:** Beppe Benedetto Consentino, Giuseppe Virga, Gaetano Giuseppe La Placa, Leo Sabatino, Youssef Rouphael, Georgia Ntatsi, Giovanni Iapichino, Salvatore La Bella, Rosario Paolo Mauro, Fabio D’Anna, Teresa Tuttolomondo, Claudio De Pasquale

**Affiliations:** 1 Department of Agricultural, Food and Forest Sciences, University of Palermo, 90128 Palermo, Italy; beppebenedetto.consentino@unipa.it (B.B.C.); giuseppe251@hotmail.it (G.V.); gaetanoglaplaca@gmail.com (G.G.L.P.); giovanni.iapichino@unipa.it (G.I.); fabio.danna@unipa.it (F.D.); teresa.tuttolomondo@unipa.it (T.T.); claudio.depasquale@unipa.it (C.D.P.); 2 Department of Agricultural Sciences, University of Naples Federico II, 80055 Portici, Italy; youssef.rouphael@unina.it; 3 Laboratory of Vegetable Production, Department of Crop Science, Agricultural University of Athens, 11855 Athens, Greece; ntatsi@aua.gr; 4 Dipartimento di Agricoltura, Alimentazione e Ambiente (Di3A), University of Catania, via Valdisavoia, 5-95123 Catania, Italy; rosario.mauro@unict.it

**Keywords:** *Apium graveolens* L., plant-derived protein hydrolysates, animal-derived protein hydrolysates, sustainable vegetable production, mineral composition, functional features

## Abstract

The vegetable production sector is currently fronting several issues mainly connected to the increasing demand of high quality food produced in accordance with sustainable horticultural technologies. The application of biostimulants, particularly protein hydrolysates (PHs), might be favorable to optimize water and mineral uptake and plant utilization and to increase both production performance and quality feature of vegetable crops. The present study was carried out on celery plants grown in a tunnel to appraise the influence of two PHs, a plant-derived PH (P-PH), obtained from soy extract and an animal PH (A-PH), derived from hydrolyzed animal epithelium (waste from bovine tanneries) on yield, yield components (head height, root collar diameter, and number of stalks), mineral composition, nutritional and functional features, as well as the economic profitability of PHs applications. Fresh weight in A-PH and P-PH treated plants was 8.3% and 38.2% higher, respectively than in untreated control plants. However, no significant difference was found between A-PH treated plants and control plants in terms of fresh weight. Head height significantly increased by 5.5% and 16.3% in A-PH and P-PH treated plants, respectively compared with untreated control (*p* ≤ 0.05). N content was inferior in PHs treated plants than in untreated control. Conversely, K and Mg content was higher in A-PH and P-PH treated plants as compared to the untreated ones. Furthermore, A-PH and P-PH improved ascorbic acid content by 8.2% and 8.7%, respectively compared with the non-treated control (*p* ≤ 0.001). Our results confirmed, also, that PHs application is an eco-friendly technique to improve total phenolic content in celery plants. In support of this, our findings revealed that animal or plants PH applications increased total phenolics by 36.9% and 20.8%, respectively compared with untreated plants (*p* ≤ 0.001).

## 1. Introduction

*Apium graveolens* L. is a vegetable belonging to the *Apiaceae* family. It originates from the Mediterranean area of Southern Europe and from the marshlands of Egypt and Sweden. *Apium graveolens* L. includes three cultivated taxonomic varieties: Celery (var. *dulee*), celeriac (var. *rapaeeum*), and smallage (var. *secalinum*) [1,2].

Celery has a long fibrous stalk tapering into leaves. Depending on location and cultivar, either its stalks, leaves or hypocotyl are edible and used as a vegetable, whereas its seeds are used for spicing and for medicinal purposes [3,4,5]. The leaves and stalks (petioles) are consumed mainly raw in salad or cooked in soups. Celery comprises different health promoting constituents such as, dietary fiber, vitamins, minerals, and amino acid tryptophan [3]. Furthermore, some authors [6] found that celery contains compounds such as 3-n-butylphthalide and the related phathalide and sedanolide, which enhance the action of glutathione-s-transferase in the liver and in the small intestinal mucosa.

Nowadays, the vegetable crop sector is aimed to satisfy the increasing vegetable demand by boosting sustainable agronomic practices, improving crop productivity and ameliorating fruit quality [7].

Plant biostimulants are a group of compounds and/or microorganisms which represent a novel sustainable tool to enhance plant performance, yield and fruit quality under stressful or no-stressful cultivation conditions [8,9,10,11,12,13,14,15,16,17]. Rouphael et al. [18] and Rouphael and Colla [19] found that biostimulants ameliorate plant responses, such as nutrient use efficiency (NUE), stress tolerance, fruit features and nutrient uptake. In addition, there are reports of a progressive influence of plant biostimulants on functional features of fruiting and green leafy vegetables such as tomato [11], pepper [20], spinach [21] and wall rocket [22] was observed. 

Plant biostimulants can be provided via fertigation and/or foliar spray. In the latter case, these compounds are engaged by cuticle, epidermal cells and stomata, and lastly achieve the cells of the mesophyll [23]. Protein hydrolysates (PHs) are defined as combination of free amino acids or oligo- and polypeptides resulting by chemical (acid and alkaline hydrolysis), enzymatic or chemical-enzymatic hydrolysis of plant rests or animal tissues [24]. PHs, derived from enzymatic hydrolysis procedure, are environmentally friendly [15] and, consequently, suitable for organic farm management [25]. Numerous researches have confirmed that PHs stimulate plant growth, development and yields due to hormone-like activities such as, auxin and gibberellins [26]. Furthermore, there are reports that PHs ameliorate the minerals uptake, assimilation and translocation via the alteration of the roots in respect to biomass, root density and lateral root branching [27,28,29]. To the best of our acquaintance, there are no reports on the effects of animal-derived PH (A-PH) and plant-derived PH (P-PH) on celery quantitative and qualitative features. Thus, starting from the aforesaid considerations, the aim of our study was to appraise the type of PH (animal or plant-derived) most suited to improve yield, nutraceutical traits and mineral content as well as economic profitability of celery cultivated in a protected environment.

## 2. Results

### 2.1. Morphological Features and Yield

Morphological features and yield are reported in Table 1. P-PH significantly affected head fresh weight; the highest values were recorded in plants treated with P-PH, followed by those from plants treated with A-PH and untreated plants.

Plants subjected to the P-PH treatment gave the highest head height, whereas untreated plants gave the lowest one. However, plants from plots treated with A-PH did not significantly differ neither from untreated plants nor from those treated with P-PH. PHs did not significantly affect root collar diameter and number of stalks.

### 2.2. Mineral Composition and Functional Features

Mineral composition of celery plants is presented in Table 2. PHs significantly influenced N content; the highest concentration was detected in the non-treated plants, whereas the lowest were found in plants treated with P-PH. PHs treatments did not significantly affect P and Ca ions concentration in celery plants.

PHs-treated plants had the highest concentration of K ions, whereas, control plants had the lowest. Data collected on concentration of Mg ions supported the trend established for concentration of K ions. 

Data on functional features are reported in Table 3. PHs meaningfully affected percentage of head dry matter; non-treated plants gave the highest percentage of head dry matter value and PH-treated plants the lowest.

Data recorded on TA sustained the trend recognised for percentage of head dry matter. Regarding SSC, the highest value was observed in untreated plants, whereas, the lowest ones were collected from plants treated with P-PH. However, plants from plots treated with A-PH did not significantly differ neither from non-treated plants nor from those treated with P-PH. PHs treatments did not significantly affect pH in celery plants. Plants treated with A-PH and P-PH had the highest ascorbic acid content, whereas non-treated plants had the lowest.

PHs positively affected total phenolics in celery; the highest value was observed in plants from plots treated with A-PH, followed by that observed in plants treated with P-PH. The lowest values were collected from non-treated plots.

Data on stalks and leaves color are showed in Table 4. PH-treatments considerably affected color traits; as concern the stalks color, non-treated plants gave the highest a* color coordinate whereas PH-treated plants (both, P-PH and A-PH treated plants) gave the lowest. The treatments had no significant effect on stalks b* color coordinate.

Whereas, with regard to the stalks L* color coordinate, PHs-treated plants were characterized by the highest values compared with control plants. Data on leaves a* and b* color coordinates supported the trend established for stalks a* and b* color coordinates. PH-treatments did not influence leaves L* color coordinated. 

### 2.3. Principal Component Analysis (PCA)

The findings of the PCA revealed two principal components (PCs) (eigenvalues higher than 1.00) elucidating for 81.82% and 18.18% of the total variance, respectively (Table 5).

**Figure 1:**
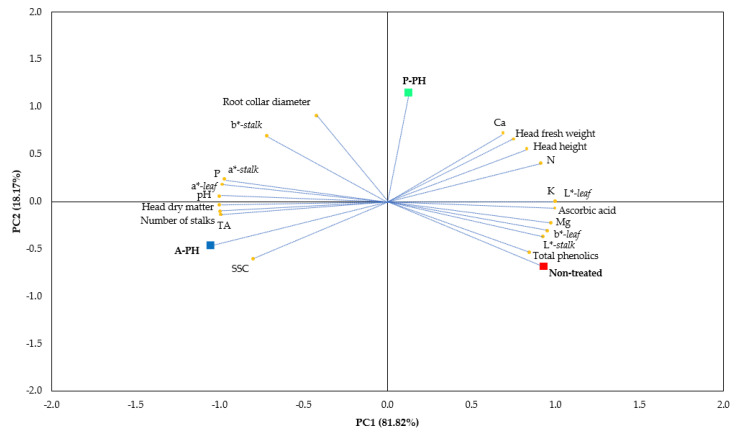
Principal component loading plot and scores of the principal component analysis (PCA) of the yield and morphological components, mineral composition (N, P, K, Ca, and Mg), head dry matter, soluble solids content (SSC), pH, titratable acidity (TA), bioactive molecules (ascorbic acid and total phenolics) and stalks and leaves color traits of celery as a function of animal or plant-derived protein hydrolysates. A-PH = animal-derived PH; P-PH = plant-derived PH. N = nitrogen; P = phosphorus; K = potassium; Ca = calcium; Mg = magnesium; SSC = soluble solid content; TA = titratable acidity.

PC1 was positively correlated to head fresh weight, head height, N, K, Ca, Mg, ascorbic acid, total phenolics, L*-stalk, b*-leaf and L*-leaf, and negatively related to number of stalks, P, head dry weight, SSC, pH, TA, a*-stalk, b*-stalk and a*-leaf; PC2 was principally positively correlated to head fresh weight, root collar diameter, Ca and b*-stalk and negatively connected to SSC (Table 5). The graphical illustration of the original variables on the plane PC1-PC2 clearly elucidate such a connection, as denoted in the plot of loadings (Figure 1).

The discrimination of the different PHs, animal or plant-derived, can be visualized in the plot of scores (Figure 1), where three clusters are noticed. The non-treated is situated on the bottom-right position, the P-PH is allocated on the top-right side, whereas, A-PH is sited on the bottom-left position (Figure 1).

### 2.4. Partial Budget Analysis of Protein Hydrolysates-Treated Celery Production 

The yield increase due to the PHs treatments is reported in Table 6. It is evident, an economical beneficial effect of the PHs treatments, 3445.8 and 15,901.7 Euro ha^−1^ respectively, with the highest added gross return recorded in celery plants treated with P-PH (Table 6). Among the considered added variable costs, PH treatment was the main cost element, followed by foliar spraying. Whereas, the harvest cost item did not undergo any change, since, in celery, it is a cost item strictly correlated to the number of plants per hectare (Table 6). Furthermore, there was an added net return of 2528.8 and 14,685.4 Euro ha^−1^ for celery plants treated with A-PH and P-PH, respectively (Table 6). Generally, our findings showed that the increased net economical benefit was linked with the yield enhancement. Particularly, using P-PH was more profitable than using A-PH. Nevertheless, A-PH gave a net economical benefit compared with the control. 

## 3. Discussion

In the near future, as reported by Searchinger [30], the vegetable production sector must face the dual challenge of nourishing an increasing world population, while reducing the impact on human well-being and on the environment. Colla et al. [29] reported that protein hydrolysates (PHs) are an advanced technology with promising application prospectives to confront with the aforesaid challenges. Our results revealed that yield enhancements can be accomplished using P-PHs. Overall our outcomes agree with those by Schiavon et al. [31] who found that, an alfalfa protein hydrolysate may enhance Zea mays productivity. There are reports of similar results on corn, kiwifruit, lily, papaya, passionfruit, and vegetables such as lettuce, pepper, and tomato [11,25,27,28,29,32]. Protein hydrolysates take affect by implementing root vigor which in turn improves water effectiveness and nutrient uptake and, consequently, crop productivity. In this regard, Colla et al, [32] observed that root dry weight and root biometric traits were superior in plants treated with PH in comparison with non-treated plants. Our results on head height are consistent with those of Matsumiya and Kubo [33], who revealed that, tomato, eggplant and Indian mustard growth are stimulated by the supply of plant growth promoting peptides derived from soybean. The better response of P-PH may be attribute to the fact that PH TAYSON® comprises tryptophan, a precursor of indole-3-acetic acid which is responsible for the expansion of shoots and roots. Conversely, since A-PH are produced through a chemical hydrolysis, that occurs at temperatures above 121 °C, thermolabile amino acids, such as tryptophan, are degraded [34]. Our outcomes on plant N concentration are in accord with those reported by Amr and Hadidi [35], who found that the application of PHs on leafy green vegetables decreases nitrates accumulation. Liu and Lee [36] reported that plants treated with a mix of amino acids considerably decreased nitrate buildup in a number of leafy green vegetables (nitrogen iper-accumulators) such as, rocket, lettuce, swiss chard, and spinach. Furthermore, Tsouvaltzis et al. [37] found that nitrate accumulation decreases as the PH “Amino 16” dosage increases. As reported by Calvo et al. [9] and Colla et al. [29], the PHs capacity in barring the high accumulation of nitrates in plants might be ascribed to the high regulation capacity of numerous metabolic pathways implicated in nitrogen metabolism. Moreover, Colla et al. [29] reported that PHs characterized by a high concentration of free amino acids lead to strong phloem loading with amino acids, which, consequently, limit root nitrate uptake and accumulation. Moreover, since in their study nitrogen content was reduced by P-PH, it was suggested that PHs stimulate celery plants to utilize its own nitrogen deposits without ex novo nitrogen uptake from the growth site. 

Our findings on mineral composition showed that PHs did not significantly affect P and Ca concentration in celery plants, conversely, A-PH and P-PH were equally effective in increasing K and Mg plant concentrations. These results tie well with a previous study [13] wherein by studying the effects of foliar application of a legume-derived PH on quantitative and qualitative features in different tomato cultivars grown in a protected environment, it was found that the application of legume-derived PH “Trainer®” significantly increased K and Mg content in tomato fruits. Furthermore, our results are in line with those of Colla et al. [11], who described similar results in another tomato experiment applying the identical PH. Our findings agree also, with those of Giordano et al. [38], who examining stand-alone and interactive influences of plant-based biostimulants on the yield and quality of perennial wall rocket, revealed that PH and tropical plant extract alone or in combination do not significantly affect P leaf content, whereas, both plant-based biostimulants tested were effective in enhancing K and Mg leaf content. Nevertheless, our findings are not directly in line with previous results reported by Caruso et al. [22], who by investigating the influences of PH or plant extract-based biostimulants on yield and quality traits of wall rocket found that the biostimulant applications did not significantly affect K and Mg concentrations. Whereas both, PH and plant extract-based biostimulants, were equally effective in increasing P and Ca contents. Accordingly, we may suppose that plant species is a critical aspect for the recognition of a proper PH biostimulant as significant variances may occur among diverse species and varieties.

Our results obtained on dry matter percentage are in contrast with those of Caruso et al. [22], who claimed that biostimulant application (both, PH or plant extract-based biostimulants) improve the leaf dry residue compared to the non-treated control. Furthermore, our findings also differ from those of Rouphael et al. [17], who studying the influences of protein hydrolysate-based biostimulant on nutritional features of greenhouse grown spinach, found that PH do not significantly affect leaf dry matter. The different response may be attributed to diverse genotype tested. In our study, PHs application enhanced ascorbic acid and total phenolics. This is consistent with the reports by Rouphael et al. [13] and Colla et al. [11], who found that foliar application of a legume-derived PH increase soluble solids, ascorbic acid and general functional quality of the tomato fruits. Since, our outcomes revealed that A-PH and A-PH reduced SSC, we may speculate that SSC is a trait highly related to the genotype. Our results also agree with those of Caruso et al. [22], who showed that PH positively affect ascorbic acid and polyphenols in wall rocket. Furthermore, our findings are consistent with those by Gurav and Jadhav [39], who detected a significant improvement in total phenolics when banana plants where fed with feather degradation products comprising both amino acids and peptides. This suggests that the application of PHs increases plants photosynthetic activity and, consequently, promotes secondary metabolism [20,26]. Moreover, according to Rouphael et al. [40], the increase of ascorbic acid might related to the augmented nutrient assimilation (of both macro- and micronutrients) of PH-treated plants, which could emphasize the production of some amino acids such as tyrosine and phenylalanine. 

Nitrogen is damaging to human health since it can cause gastric cancer and further illnesses [9]. Our results showed that plants treated with P-PH performed better than plants treated with A-PH in terms of head fresh weight and nitrogen concentration. In this respect, we might hypothesize that, as reported by Bonner and Jensen [41], a phenomenon such as the “general amino acid inhibition” might be occurred. In support of this, our results displayed that A-PH positively affected the total phenolics compared with untreated plants or treated with P-PH. This highlights that some stressful conditions, due to the A-PH treatment, encourage an increase of phenolics [42,43,44,45]. Thus, we could speculate that the higher total phenolic level is due to a distress triggered by a non-optimal condition caused by the A-PH treatment. Indeed, A-PHs, produced via a chemical hydrolysis, have a higher salinity compared with the P-PHs produced though enzymatic hydrolysis, due to the alkali content, such as sodium, potassium or calcium hydroxide, and acids like hydrochloric or sulfuric acid [34]. Our outcomes on color traits revealed that PH treatments (plant or animal-derived), enhanced the greenish of stalks and leaves. Moreover, PH treatments significantly improved the lightness of the stalks. Our results are in contrast with those of Caruso et al. [22] and Giordano et al. [38], who found that tropical plant extract or legume-derived PH did not significantly affect plant color coordinates in wall rocket, although, a positive effect in terms of SPAD was declared. These conflicting findings could be associated to the dissimilar PHs origin, PH production process, genotype, application dose, and environmental growing conditions.

## 4. Materials and Methods 

### 4.1. Experimental Site, Growing Conditions, and Biostimulant Treatments

**Figure 2:**
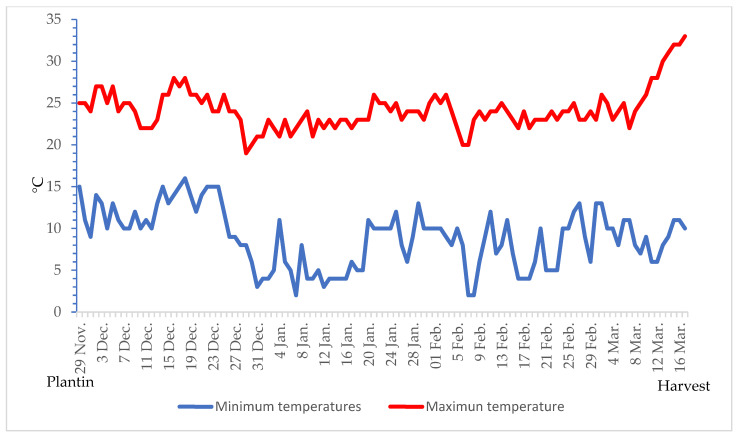
Minimum and maximum air temperature inside a tunnel located in Marsala, Trapani province (longitude 12°26’ E, latitude 37°47’ N, altitude 37 m) during the celery growing cycle.

The research was conducted during the 2020 winter-spring season, in an organic farm located in Marsala, Trapani province of Sicily (longitude 12°26’ E, latitude 37°47’ N, altitude 37 m). The experiment was performed in a tunnel (30.0 m in length, 5.0 m in width and 2.0 m in height), open on both sides, and covered with a transparent polyethylene (PE) film. The soil employed for the research was originated from Sicilian “sciare” soil transformation. It was, essentially, characterized by sand (<80%) at pH 8.5, high activity limestone at 8.8% and richly endowed with exchangeable K_2_O (660 mg kg^−1^), phosphorous (68 mg kg^−1^), total nitrogen (2%), and organic matter (10 t·ha^−1^). Throughout the whole experiment, the soil was mulched with a black PE film (0.20 µm) and a drip irrigation system was installed. Maximum and minimum daily temperatures were recorded using a data logger (Figure 2).

“Malachit” F_1_ (RBsementi, Torin, Italy) celery (var. *dulee*) plugs were transplanted on 29 November 2019, spacing 0.40 m between rows and 0.30 m inter-rows, obtaining a plant density of 8.3 plant·m^−2^. During the growing period, all celery needs were satisfied following the cultivation practices recommended for an organic management. Briefly, aged manure was added to the soil in pre-transplant (40 t ha^−1^). After transplant, all plots received a fertigation dose of nitrogen 150 kg ha^−1^, potassium oxide 150 kg ha^−1^ and sulfur trioxide 115 kg ha^−1^. All plots were equally irrigated via a drip irrigation system. A plant PH (TYSON^®^, Mugavero fertilizers, Palermo, Italy), obtained from soy extract and an animal PH (ASWELL^®^, Mugavero fertilizers, Palermo, Italy), derived from hydrolyzed animal epithelium, were tested. TYSON^®^ is an organic biostimulant rich in nitrogenous elements deriving completely from amino acids and plant peptides, obtained via the use of specific enzymes at low temperatures. It is a product containing a mixture of nitrogen-rich constituents highly soluble and easily assimilated by plants (Table 7). 

ASWELL^®^ is an organic nitrogen fertilizer with a high supply of low molecular weight free amino acids (10%), with functions of nutritional balancer and phytostimulant for foliar and radical applications (Table 8).

The PHs application started one week after transplant. Weekly, biostimulant solutions or water (control treatment) were supplied via foliar spray, rendering a total of 14 applications. For both biostimulants the recommended dose (3 mL·L^−1^) was adopted. Plants were harvested on 17 March, 2020.

### 4.2. Yield and Yield Related Traits, Nutrient and Functional Features

Proximately after harvest, head fresh weight (g), head height (cm), number of stalks and collar diameter (mm) were measured on 5 plants randomly selected from each replicate. Head dry weight was determined by drying the sample in a thermo-ventilated oven (M40-VF, Artiglass, Padova, Italy) at 105 °C till constant mass. 

Analyses on nutrient and functional traits were carried out with three replicates. For the determination of soluble solids content (SSC), a sample (stalks + leaves) of 100 g for each plant was juiced and filtered. The SSC was recorded via a refractometer (MTD-045 nD, Three-In-One Enterprises Co. Ltd. New Taipei, Taiwan).

On ten undamaged leaves per replicate, stalk and leaf color coordinates–CIELab (a*, b* and L*) were measured by a colorimeter (Chroma-meter CR-400, Minolta Corporation, Ltd., Osaka, Japan).

Samples for mineral determinations were composed at harvest. For nitrogen (N) determination, the Kjeldal method was adopted. 

For Ca, Mg, and K, determinations the official methods reported by Morand and Gullo [46] were applied. Leaf phosphorus content (P) was evaluated using colorimetry [47].

Titratable acidity (TA) was assessed by titrating 10 mL of juice with NaOH 0.1N to an endpoint of pH 8.1 and expressed as g of citric acid 100 mL^−1^. The pH was appraised with the same equipment used for measuring TA.

Ascorbic acid content was measured from the leaf samples by reflectometer Merck RQflex* 10 m using Reflectoquant Ascorbic Acid Test Strips. The results were expressed as g·100 mL^−1^ of juice.

For total phenolics determination, samples of 5 g were extracted using methanol and was analysed quantitatively by A760. Total phenolics content was measured as described by Singleton and Rossi [48]. The outcomes were expressed as mg of catechin/100 g dry matter.

### 4.3. Experimental Design and Statistical Analysis

The experiment was organized in a randomized complete block design (RCBD) with three blocks per treatments. The experiment includes 3 treatments consisting of 60 plants for each treatment, rendering 180 plants. The statistical analysis was performed with the software SPSS package version 20.0 (StatSoft, Inc., Chicago, IL, USA) using One-Way Analysis Of Variance (ANOVA). Data expressed as percentage underwent angular transformation (Ø = arcsin (p/100)^1/2^) prior to ANOVA. For means separation, Tukey’s HSD test (*p* < 0.05) was adopted. Principal component analysis (PCA) was accomplished on the whole yield, nutritional and functional properties. For optimal number of principal component selection (PCs), factors with eigenvalues higher than 1.0 were considered. The initial variables were projected into the subspace demarcated by the reduced number of PCs and associated parameters were acclaimed.

### 4.4. Partial Budget Analysis

The partial budget analysis was drawn up to appraise the net economic profits that may accrue to the celery growers employing the PHs. We used the economic method described by Giordano et al. [39]. For the two PHs, the added costs and gross returns by using the PHs compared to the control treatment were considered. To calculate the added net return sustained by both A-PH and P-PH, the subsequent formula was employed: Added net return = added gross return − added variable costs.

## 5. Conclusions

In the present work, PHs treatments significantly affected yield and yield related traits, mineral composition, and functional features in celery. P-PH effectively influenced plant fresh weight and head height, whereas, both P-PH and A-PH, affected plant N content, K and Mg concentrations, as well as, ascorbic acid and total phenolics. Our results also showed that PHs treatments increased stalks and leaves greenish and improved stalks lightness. Finally, plants subjected to P-PH performed better than plants treated with A-PH in terms of head fresh weight and nitrogen concentration. However, our findings suggest that both, P-PH “TYSON^®^” (obtained from soy extract) and A-PH “ASWELL^®^” (derived from hydrolyzed animal epithelium), may effectively enhance crop performance, nutritional and functional features as well as the economic profitability of celery.

## Figures and Tables

**Table 1:** Effect of different protein hydrolysates (PHs) on head fresh weight, head height, root collar diameter, and number of stalks of celery.

Treatment	Head Fresh Weight (g)		Head Height (cm)		Root Collar Diameter (mm)		Number of Stalks (no.)	
Non-treated	713.33	b	52.60	b	35.00	a	17.17	a
A-PH	772.40	b	55.50	ab	29.50	a	15.83	a
P-PH	985.93	a	61.17	a	34.83	a	15.33	a
Significance	**		*		NS		NS	

Data within a column followed by the different letter are significantly different at *p* ≤ 0.05. NS, *, ** non-significant or significant at 0.05 and 0.01, respectively. A-PH = animal-derived PH; P-PH = plant-derived PH.

**Table 2:** Effect of different PHs on nitrogen (N), phosphorus (P), potassium (K), calcium (Ca), and magnesium (Mg) plant concentrations (mg g^−1^ dw) of celery.

Biostimulant	N		P		K		Ca		Mg	
Non-treated	5.29	a	1.13	a	3.38	b	1.23	a	0.63	b
A-PH	5.08	b	1.04	a	4.24	a	1.24	a	0.80	a
P-PH	4.89	c	1.05	a	4.37	a	1.31	a	0.78	a
Significance	***		NS		***		NS		***	

Data within a column followed by the different letter are significantly different at *p* ≤ 0.05. NS, *** non-significant or significant at 0.001, respectively. A-PH = animal-derived PH; P-PH = plant-derived PH.

**Table 3:** Effect of different PHs on head dry weight, soluble solid content (SSC), pH, titratable acidity (TA), ascorbic acid and total phenolics of celery.

Biostimulant	Head Dry Matter (%)		SSC (Brix°)		pH		TA (g citric acid 100 mL^−1^)		Ascorbic Acid (g 100 mL^−1^)		Total Phenolics (mg of catechin 100 g^−1^ dw)	
Non-treated	9.28	a	4.97	a	6.39	a	0.73	a	5.62	b	3.36	c
A-PH	7.52	b	4.77	ab	6.34	a	0.63	b	6.08	a	4.60	a
P-PH	7.00	b	4.23	b	6.33	a	0.61	b	6.11	a	4.06	b
Significance	*		*		NS		**		***		***	

Data within a column followed by the different letter are significantly different at *p* ≤ 0.05. NS, *, **, *** non-significant or significant at 0.05, 0.01 and 0.001, respectively. A-PH = animal-derived PH; P-PH = plant-derived PH.

**Table 4:** Effect of different PHs on stalks and leaves color coordinates of celery.

Treatment	Stalks						Leaves					
	a*		b*		L*		a*		b*		L*	
Non-treated	−16.02	a	34.54	a	52.26	b	−15.66	a	29.52	a	44.43	a
A-PH	−19.51	b	29.73	a	58.44	a	−18.74	b	31.95	a	50.55	a
P-PH	−19.05	b	32.68	a	56.79	a	−18.95	b	31.46	a	51.48	a
Significance	**		NS		**		*		NS		NS	

Data within a column followed by the different letter are significantly different at *p* ≤ 0.05. NS, *, ** non-significant or significant at 0.05 and 0.01, respectively. +a* (redness); −a* (greenness); +b* (yellowness); −b* (blueness); L* (lightness). A-PH = animal-derived PH; P-PH = plant-derived PH.

**Table 5:** Correlation coefficients for 21 parameters, eigenvalues, variance and cumulative proportions of total variance of the two principal components (PCs).

Variable	PC1	PC2
Head fresh weight	**0.750**	**0.661**
Head height	**0.830**	0.558
Root collar diameter	−0.424	**0.906**
Number of stalks	**−0.990**	−0.139
N	**0.915**	0.404
P	**−0.983**	0.183
K	**1.000**	0.003
Ca	**0.690**	**0.724**
Mg	**0.975**	−0.224
Head dry matter	**−0.995**	−0.100
SSC	**−0.799**	**−0.601**
pH	**−0.990**	−0.139
TA	**−0.999**	−0.039
Ascorbic acid	**0.998**	−0.069
Total phenolics	**0.845**	−0.535
a*-*stalk*	**−0.971**	0.238
b*-*stalk*	**−0.717**	**0.697**
L*-*stalk*	**0.929**	−0.370
a*-*leaf*	**−0.998**	0.060
b*-*leaf*	**0.952**	−0.305
L*-*leaf*	**1.000**	0.004
Eigenvalue	17.183	3.817
Variance %	81.823	18.177
Cumulative %	81.823	100.000

Values in bold are the variables with the largest correlation. N = nitrogen; P = phosphorus; K = potassium; Ca = calcium; Mg = magnesium; SSC = soluble solid content; TA = titratable acidity.

**Table 6:** Added returns acquired by animal-derived protein hydrolysates (A-PH) and plant-derived protein hydrolysates (P-PH) under tunnel conditions compared to the untreated control.

Protein Hydrolysate	Yield Increase (t ha^−1^)	Price (€ t^−1^)	Added Gross Return (€ ha^−1^)	Added Variable Cost (€ ha^−1^)				Added Net Return (€ ha^−1^)
				PH Treatment	Foliar Spraying	Harvest	Total	
A-PH	4.9	700.0	3445.8	567.0	350.0	0.0	917.0	2528.8
P-PH	22.7	700.0	15901.7	866.3	350.0	0.0	1216.3	14685.4

Costs of protein hydrolysates were provided by suppliers (TYSON^®^ = 8.25 €/kg; ASWELL^®^ = 5.40 €/kg); costs of foliar spraying were considered established on the information provided by local agricultural contractors.

**Table 7:** Composition of P-PH tested (TYSON®, Mugavero fertilizers, Italy, Palermo).

Component	Percentage Composition (%)
Total nitrogen	5.0
Organic nitrogen	4.5
Organic carbon	25.0
Amino acids	31.0

P-PH = plant-derived PH.

**Table 8:** Composition of A-PH tested (ASWELL®, Mugavero fertilizers, Palermo, Italy).

Component	Percentage Composition (%)
Total nitrogen	8.0
Organic nitrogen	7.7
Ammoniacal nitrogen	0.3
Amino nitrogen	2.8
Amino acids	48.2
Free amino acids	10.0
Organic carbon	22.6

A-PH = animal-derived PH.
